# Counting‐based cell‐free DNA screening test fails to identify triploidy—A case report

**DOI:** 10.1002/ccr3.1812

**Published:** 2018-11-19

**Authors:** Ji E. Park, Ji K. Park, Min Y. Kang, In A. Cho, Jong C. Baek

**Affiliations:** ^1^ Department of Obstetrics and Gynecology College of Medicine Gyeongsang National University Gyeongsang National University Changwon Hospital Changwon Korea; ^2^ Department of Obstetrics and Gynecology College of Medicine Gyeongsang National University Hospital Jinju Korea

**Keywords:** 69,XXX, noninvasive prenatal testing, ultrasound

## Abstract

Although noninvasive prenatal testing (NIPT) is a good test with high sensitivity and specificity for trisomy 21, 18, and 13, it remains a screening test and cannot be used for diagnostic purposes. It is important to consider the outcomes of this test and interpret the results and offer consultation accordingly.

## 1 INTRODUCTION

The results of the combined test indicated the pregnancy was at high risk of Edwards syndrome. The counting‐based noninvasive prenatal test showed a low risk of trisomy 21, 18, and 13, and the patient was relieved of chromosomal abnormalities. We identified 69,XXX in the amniocentesis after fetal ultrasound anomalies.

Noninvasive prenatal testing (NIPT) using cell‐free DNA from maternal plasma is an increasingly popular option for prenatal screening, owing to its improved performance over traditional screening techniques. Since its introduction, prenatal screening for commonly observed aneuploidies has rapidly been adopted clinically.^1^ Although it has high clinical sensitivity and specificity for trisomy 21, some other, rarer cytogenetic conditions are less likely to be detected with some NIPT methods. Triploidy is a rare genetic condition and most cases usually end in spontaneous first trimester abortions.^2^ Here, we report a case of triploidy that could not be diagnosed using counting‐based NIPT. This case emphasizes the necessity for adequate pre‐ and post‐test genetic counseling to ensure that patients receive an appropriate prenatal diagnosis and are educated about prevalent NIPT.

## 2 CLINICAL REPORT

### 2.1 Case description

A 37‐year‐old nulliparous woman was referred for detailed fetal sonographic evaluation for heart anomalies and growth restriction at 20 and 5/7 weeks of gestation. She denied using teratogenic medications, recent viral infection, diabetes mellitus, and hypertension. She and her husband were nonconsanguineous and appeared healthy. There was no family history of congenital malformation.

The results of the stepwise sequential screening test [pregnancy‐associated plasma protein (PAPP)‐A 0.056 multiples of the median (MoM), free beta human chorionic gonadotropin (hCG) 0.074 MoM, nuchal translucency (NT) 0.874 MoM, α‐fetoprotein (AFP) 0.616 MoM, hCG 0.052 MoM, unconjugated estriol (uE3) 0.107 MoM, and inhibin‐A 0.303 MoM] indicated that the fetus of the 37‐year‐old mother was at high risk of Edwards syndrome (1:5). The mother was offered amniocentesis, but declined the invasive diagnostic test in favor of a noninvasive option, Faest^©^ NIPT (Macrogen, Seoul, Korea), at 17 and 6/7 weeks of gestation. Faest^©^ is a NIPT protocol based on massively parallel shotgun whole genome sequencing. The quantity of the fragments from each chromosome is assessed and compared with that of controls, and this comparison is then used to screen for trisomy 21, 18, and 13. When the results were reported as “low risk of trisomy 21, 18, and 13,” the parents and physician concluded that these results were reassuring news that the fetus was negative for any chromosomal anomaly, instead of just the trisomies that are currently screened for by this test (trisomies 21, 18, and 13).

**Figure 1:**
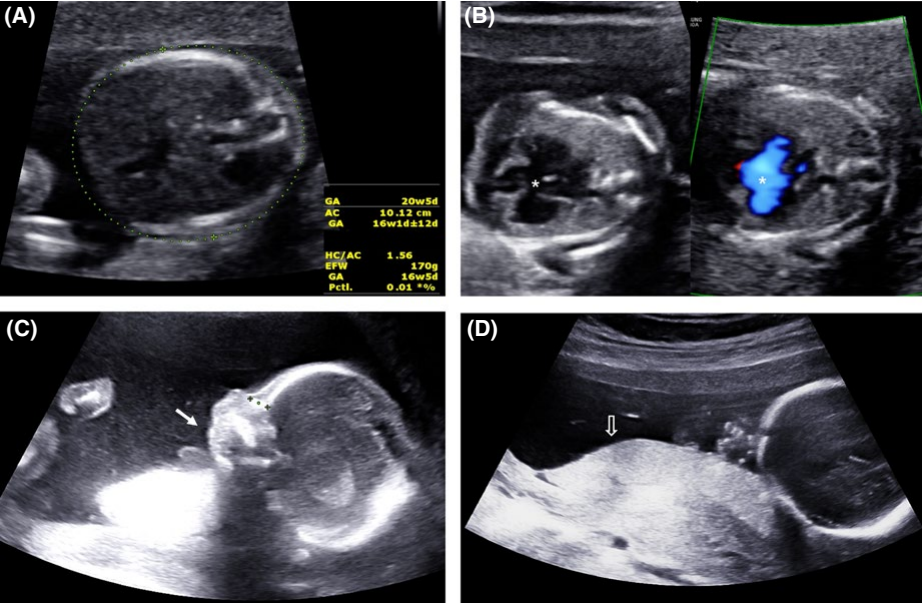
Ultrasonographic findings at 20 wk, 5 d of gestation. A, An abdominal circumference smaller by 4 wk of gestational age. B, A complete atrioventricular canal defect (asterisk). C, Micrognathia (arrow). D, Placenta (open arrow)

A fetal ultrasound performed at our hospital indicated micrognathia, a complete atrioventricular canal defect, and small‐for‐gestational‐age; biometric data on the head and abdomen were discordant with the age of gestation by 2 and 4 weeks, respectively (Figure 1). The amniotic fluid remained normal.

After extensive parental counseling, the parents agreed to undergo amniocentesis to confirm the chromosomal anomaly.

### 2.2 Cytogenetic and FISH analysis

**Figure 2:**
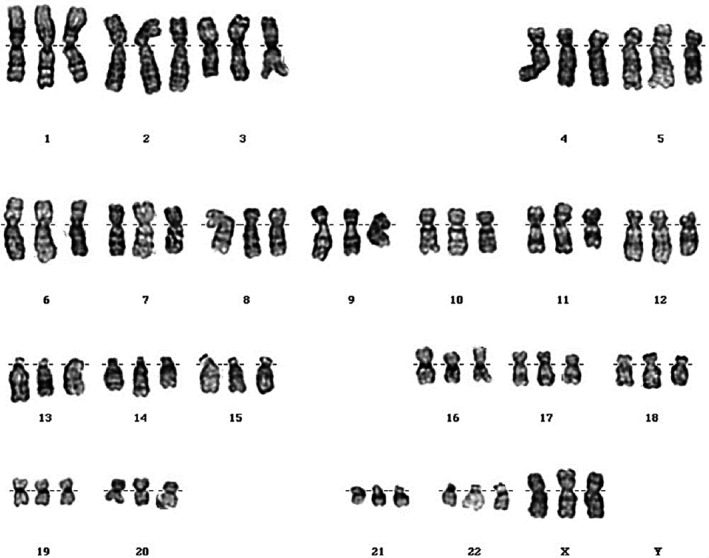
Karyotype of the fetus from amniotic fluid showing 69,XXX

At 20^+5^ weeks of gestation, conventional karyotyping and fluorescence in situ hybridization (FISH) analyses were performed using cells obtained from amniotic fluid. Conventional GTG‐banding analysis was carried out with cultured amniocytes. All 20 metaphase chromosomes from the amniotic fluid sample indicated that the fetus had a triploid chromosome (Figure 2). We reported the fetal karyotype as 69,XXX. FISH analyses were performed using TUPLE1 region probes, which map to 22q11.2, and the ARSA control probe, which maps to 22q13. Of the interphase chromosomes from the amniotic fluid sample, 196 of 200 indicated that the fetus had gain of the TUPLE (22q11.2) and ARSA (22q13) regions.

After extensive parental counseling, the parents decide to terminate the pregnancy at a local clinic.

## 3 DISCUSSION

Triploidy, in which the fetus has three copies of every chromosome, accounts for approximately 1% of recognized conceptions. Most of these fetuses are aborted spontaneously during the first trimester. The prevalence of triploidy at 12 weeks of pregnancy is 1 in 2000 and falls to 1 in 250 000 by 20 weeks.^3^, ^4^ There are two triploidy phenotypes, depending on whether the extra haploid set is paternal (diandric) or maternal (digynic). The digynic type is characterized by severe fetal growth restriction with a small placenta, normal fetal NT thickness, and very low serum free β‐human chorionic gonadotropin (β‐hCG) and pregnancy‐associated plasma protein A (PAPP‐A). In the diandric type, the placenta is usually enlarged and accompanied by a partial mole. Fetal growth restriction is not severe, fetal NT tends to be increased, and maternal serum‐free β‐hCG is approximately 10 times higher than normal. Diandric triploidy can cause severe maternal complications, including severe early‐onset preeclampsia and choriocarcinoma.^5^, ^6^, ^7^ In this case, the NT of the fetus was in the normal range and β‐hCG and PAPP‐A were low, which was phenotypically appropriate for the digynic type.

The majority of clinical NIPT methods use a quantitative counting approach that relies on comparing the absolute number of sequence reads from the chromosome of interest with reference chromosome(s), and fetal trisomy is inferred when this ratio exceeds a predetermined threshold. Since all chromosomes in triploid fetuses are trisomic, the ratio is identical to that of euploid fetuses; the requirement for a reference chromosome with this method precludes triploidy detection. This approach cannot determine the source of the DNA (fetal or maternal) and is therefore unable to detect additional fetal haplotypes associated with triploidy or vanishing twins. Vanishing twins accounted for 15% of false positives in counting‐based NIPT studies.^8^, ^9^, ^10^

The single nucleotide polymorphism (SNP)‐based approach examines the relative distributions of different alleles at polymorphic loci and does not require a reference chromosome. Therefore, it has the unique ability to detect the presence of additional fetal haplotypes associated with dizygotic twins and triploidy. This approach identifies the presence of additional fetal haplotypes, indicative of a triploid or dizygotic multifetal pregnancy, and determines parental origin.^11^, ^12^ However, this method currently does not distinguish between these possibilities. In a recent study, SNP‐based NIPT (Panorama Prenatal test; Natera) correctly identified all four diandric trisomy cases, while all four digynic cases tested were found to have low cell‐free fetal DNA (cffDNA) fractions after adjusting for maternal weight and gestational age (<4%) and, consequently, escaped diagnosis. The small size of the placenta in digynic triploidy probably contributes to the low cffDNA.^11^ There is a limit to the ability of current technology to screen digynic triploidy, and further research on the development of highly sensitive NIPT technology is needed. At present, as in this case, it is reasonable to perform invasive diagnostic testing when digynic triploidy is suspected.

Several professional organizations, including the American College of Obstetricians and Gynecologists (ACOG), the Society of Maternal Fetal Medicine (SMFM), and the International Society of Ultrasound in Obstetrics and Gynecology (ISUOG), have released position statements to help guide prenatal practice on the indications for the use of NIPT.^13^, ^14^, ^15^ If a fetal structural anomaly is identified on ultrasound examination, invasive prenatal diagnosis should be offered.^13^ A recent report stated that NIPT should not be recommended for the genetic evaluation of the etiology of ultrasound anomalies, as both the resolution and sensitivity, or negative predictive value, are inferior to those of conventional karyotyping and microarray analysis. The role of NIPT as an alternative to standard invasive testing in women considered to be at very high risk (>1:10) after combined screening, but with no ultrasound anomaly, should be evaluated in prospective studies. Expert opinion currently suggests that NIPT should not replace invasive testing in this group.^15^

Overall, NIPT should not replace invasive prenatal testing in those at very high risk (as in our case, Edwards syndrome 1:5) after combined screening or with fetal ultrasound anomalies. NIPT should be offered only as a screening method and not as a diagnostic tool; it should be offered specifically for trisomies and X monosomy. In our case, NIPT was offered based on the mother's refusal of a more invasive test. This suggests a bias in maternal counseling as NIPT should not replace invasive testing. We demonstrate the importance of appropriate pre‐ and post‐test genetic counseling to ensure that prenatal patients can make informed decisions and are instructed correctly about the benefits and limitations of NIPT.

## CONFLICT OF INTEREST

None declared.

## AUTHOR CONTRIBUTIONS

JEP: analysis and interpretation of data, involved in drafting the manuscript and revising it, final approval of the version to be published. JKP: conception and design, involved in drafting the manuscript and revising it, final approval of the version to be published. MYK: analysis and interpretation of data. IAC: acquisition of data, analysis and interpretation of data. JCB: conception and design, analysis and interpretation of data.
